# Administration Technique of Intranasal Corticosteroid Sprays Among Nepali Pharmacists: Cross-Sectional Study

**DOI:** 10.2196/83042

**Published:** 2026-01-29

**Authors:** Amar Prashad Chaudhary, Suraj Kumar Thakur, Shiv Kumar Sah

**Affiliations:** 1 Tribhuvan University Teaching Hospital, MaharajgunjKathmandu, 0977, Nepal, 977 9860329145; 2 Institute of Medicine, Maharajgunj Medical Campus, Tribhuvan University, Kathmandu, Nepal

**Keywords:** intranasal corticosteroid spray, allergic rhinitis, device use technique, pharmacist, patient counselling, continuing pharmacy education

## Abstract

**Background:**

Allergic rhinitis is a common condition affecting up to 40% of people worldwide, with a notably high prevalence in South Asia. The primary treatment for moderate to severe allergic rhinitis is intranasal corticosteroid sprays (INCS), the use of which is typically demonstrated to patients by registered pharmacists. However, many patients do not use these sprays correctly.

**Objective:**

This study evaluated the proficiency of pharmacists in demonstrating the correct technique for using INCS and the factors contributing to proper technique.

**Methods:**

In a cross-sectional survey of 365 registered pharmacists in the Kathmandu Valley, Nepal, a trained observer used a standardized 12-step checklist to assess each pharmacist’s technique for using INCS. The 12-step checklist was created after studying international guidelines, studies conducted in Nepal, international research articles, and instructional pamphlets. Simple random sampling was done to collect the data from community pharmacies in Kathmandu district. Demographics, education, experience, previous training, and instructional materials use were recorded. A total of 12 marks were awarded for all 12 steps, with one mark given for each step. Proficiency was classified as “adequate” if more than 6 marks were obtained.

**Results:**

Out of 365 pharmacists, 239 (65.5%) were male and 126 (34.5%) were female. Overall, 216 pharmacists (59.2%) were aged 26 years or younger and 235 pharmacists (69.9%) held a diploma in pharmacy. We found that 193 (52.9%) pharmacists demonstrated inadequate technique, while only 172 (47.1%) showed adequate skill overall. However, only 22 pharmacists (6%) demonstrated all 5 critical steps. The likelihood of providing appropriate counseling on the use of INCS was significantly correlated with multiple independent factors. Those with a diploma in pharmacy had a 97% lower likelihood of providing appropriate counseling compared with those with a bachelor’s degree in pharmacy and above (*P*<.001). Pharmacists who perform counseling sessions 1‐4 times per week had 11-fold greater odds of doing so correctly compared with those who do not (*P*=.002). Pharmacists who do not use educational leaflets were 96% less likely to provide adequate counseling (P= .005) . Similarly, pharmacists under the age of 26 are 89% less likely than older pharmacists to provide adequate counseling (*P*=.001). It is interesting to note that men were found to have almost 2.3 times higher odds of providing appropriate counseling than women (*P*=.02).

**Conclusions:**

More than half of the registered pharmacists in Nepal demonstrated inadequate technique when using INCS. The inadequate patient counseling on INCS use can significantly increase the risk of adverse drug reactions and reduce the efficacy of the therapy. Thus, there is a strong need for educational interventions and policy change for improved proficiency.

## Introduction

A chronic inflammatory condition of the nasal mucosa, allergic rhinitis (AR) is brought on by immunoglobulin E–mediated responses to allergens breathed in. There are many causes of AR, including pollen, dust mites, cockroach waste, animal dander, fumes and odors, changes in environment, smoke, and certain foods or spices. The most common symptoms of AR are sneezing; stuffy nose; runny nose; itchy nose, throat, eyes, and ears; nosebleeds; clear drainage from the nose; snoring; and breathing through the mouth.

AR affects 10% to 40% of the world’s population, and its prevalence is increasing in many countries [1,2]. AR and other allergy disorders are also common in Nepal and the surrounding South Asian nations. A recent school-based study in Nepal, for example, found rhinoconjunctivitis symptoms in 28% of children [3]. AR was responsible for almost 25% of allergy illnesses in Nepal’s Gandaki Province [3]. Adolescent AR prevalence in India is estimated at 22%, whereas in adults it was found to be 11% among the general population and 33.3% in asthmatics [4,5]. Similarly, a large-scale study conducted in Europe discovered that up to 20% of the population is impacted by AR [6]. The prevalence of AR in the United States is slightly lower (7.7% in adults and 7.2% in children) [7].

Therefore, the treatment of AR is very important as it impacts daily life activities. The objective of AR treatment is to control the disease. Antihistamines, leukotriene receptor antagonists, azelastine, and intranasal corticosteroid sprays (INCS) are used for treating AR according to the Allergic Rhinitis and its Impact on Asthma guidelines 2019 [8]. Effective pharmacotherapy is crucial for symptomatic control of AR. INCS are the most potent medications for moderate to severe AR and are recommended as first-line therapy [9]. When used correctly, INCS reduce nasal congestion, rhinorrhea, sneezing, and itching by suppressing mucosal inflammation.

The most common adverse drug reactions to INCS include dyspnea, anosmia, ageusia/dysgeusia, epistaxis, and headache [10]. A study conducted at the ear, nose, and throat outpatient clinic at Aberdeen Royal Infirmary found that 15.5% reported epistaxis due to an ipsilateral hand technique [11]. Similarly, a study in Thailand discovered a 3.6 times higher risk of adverse events in patients who did not point the tip of the spray away from the nasal septum [12]. Maintaining a neutral head position and exhaling through the mouth are crucial for proper drug disposition and enhanced efficacy [13]. Therefore, using the correct technique is vital for better efficacy and a reduced risk of side effects. Standard guidelines recommend instructing patients to shake the spray, remove the dust cap, blow the nose, hold the spray bottle while pointing the tip of the nozzle up with the hand, place the index and middle finger on the pusher and the thumb at the bottom of the spray bottle, maintain a neutral head position, insert the tip slightly upward and laterally (away from the septum), close the opposite nostril, inhale gently while actuating the spray, then exhale through the mouth, wipe the nozzle with a tissue or hankerchief, and replace the cap [12,14].

However, a study conducted by Rattanawong et al [12] found that only 4% of patients performed all 12 steps, while only 29% completed all the crucial steps. Similarly, a study by Gurung et al [15] in Nepal revealed that only 7.2% of patients executed all the steps correctly, and 18.2% managed to perform all 5 critical steps accurately (blow the nose, maintain a neutral head position or slightly tilt the head forward, point the tip slightly outward away from the septum, squirt the spray into the nose while breathing in, breathe out through the mouth). A systematic review indicated that approximately 73% of patients did not receive proper advice regarding INCS [16].

Health care professionals, especially pharmacists, are responsible for counseling patients regarding the drugs they dispense. Given this context, it is essential to assess how well Nepali registered pharmacists themselves understand and can demonstrate correct INCS technique. No prior studies have examined this. By identifying gaps in pharmacist knowledge and technique, targeted interventions (eg, curriculum changes or training modules) can be designed to improve AR care. This study therefore evaluated the proficiency of registered pharmacists in Kathmandu Valley in demonstrating INCS administration and analyzed professional factors associated with adequate technique.

## Methods

### Study Design and Study Period

A cross-sectional observational study was performed from November 1, 2023, to May 28, 2024, through interviews of registered pharmacists. They answered a semistructured questionnaire containing questions about their sociodemographic information, professional details, and INCS counseling steps. STROBE (Strengthening the Reporting of Observational Studies in Epidemiology) principles were adhered to in the study’s reporting [17,18].

### Study Population and Study Site

The sample was selected from pharmacists registered at the Nepal Pharmacy Council working at community pharmacies registered at the Department of Drug Administration (DDA) in Kathmandu, Nepal. Being Nepal’s capital, Kathmandu is a heavily populated city. The respective site had a large number of community pharmacies, about 4000, with many registered pharmacists [19].

### Sampling Method

Simple random sampling of the community pharmacies in different wards of Kathmandu district, Nepal, was done using Statistical Package for Social Sciences software (version 26; IBM Corp). The details of all the registered community pharmacies were obtained from the DDA database. No ward-level sampling was performed to avoid geographical clustering.

This cross-sectional study identified potential participants from the registered pharmacists working at community pharmacies. If the community pharmacy had more than one pharmacist, one pharmacist was selected for the study randomly. If the community pharmacy was closed or the pharmacist was not available, a total of three visits were made on different dates; if a pharmacist was still not available, another pharmacy was selected based on a pregenerated reserved list of random samples. These potential participants were approached and the study’s purpose, procedures, and potential risks and benefits were explained to them. The same interviewer interviewed all the participants to overcome interobserver variability in participants’ responses.

### Sample Size

The survey study was completed using the Raosoft sample size calculator to capture the appropriate sample size [20]. A minimum of 363 samples was required for a 95% confidence interval and a 5% margin of error for the population distribution of 21,000 registered pharmacists at a 40% response distribution [21]. Thus, a total of 365 registered pharmacists participated in this study.

### Measures

After the pharmacist’s sociodemographic and professional information were obtained through interviews, the 12-step nasal spray application technique as given in Textbox 1 was demonstrated by the participant and examined by the researcher [12,13,22-24]. Each correct step was assigned 1 mark, while incorrect or missed steps were assigned 0 marks. Hence, the maximum score obtained was 12 marks. Five steps in INCS counseling (indicated in Textbox 1) were considered critical based on their impact on patient outcomes and the risk of adverse drug reactions. The median value of the total marks scored was 6.

Textbox 1. **Steps for the administration of intranasal corticosteroid sprays.**

1. Shake the spray in a vertical plane.

2. Remove the dust cap.

3. Blow the nose (critical).

4. Hold the spray bottle, pointing the tip of the nozzle up with the hand.

5. Place the index and middle finger on the pusher and the thumb at the bottom of the spray bottle.

6. Put the tip of the nozzle into one nostril and close the other side.

7. Maintain a neutral head position or slightly tilt the head forward (critical).

8. Point the tip slightly outward, away from the septum (critical).

9. Squirt the spray into the nose while breathing in (critical).

10. Breathe out through the mouth (critical).

11. Wipe the nozzle with a tissue or handkerchief.

12. Replace the cap.

### Determination of the Cutoff Score

To determine the cutoff score, a sensitivity analysis was conducted for alternate cutoffs (ie, >5 and >7). The direction and significance of the main predictors remained stable at >5 and >6, indicating robustness of the findings as shown in Table S1 in Multimedia Appendix 1. The >7 cutoff produced unstable estimates due to small cell sizes. Based on a study conducted by Kc et al [25], expert suggestions, the median value, and sensitivity analysis, more than 6 marks was established as the cutoff score. Therefore, anyone with a score higher than 6 marks was categorized as performing adequately, and anyone with marks equal to or less than 6 was categorized as performing inadequately.

### Reliability and Validity

The initial questionnaire was validated by a panel of subject experts, composed of advisors, professors, and teachers, for correctness, clarity, appropriateness, and jargon use. This validation was conducted using face validity approaches. An interrater reliability test was conducted on 15 participants and found a Cronbach α value of 0.758.

### Inclusion and Exclusion Criteria

This study only took into account pharmacists aged 18 years and above who were registered with the Nepal Pharmacy Council and employed in community pharmacies. Participants needed to have a Diploma in Pharmacy (DPharm), Bachelor of Pharmacy (BPharm) degree, Doctor of Pharmacy (PharmD) degree, or Master of Pharmacy degree. Participants needed to have a minimum of 1 year of experience. No unregistered pharmacists, pharmacy students, or interns were considered for this study.

### Data Collection Procedure

The essential information was then gathered from participants using a semistructured questionnaire administered through an in-person interview. A standardized protocol was followed during interviews. Prior to their enrollment in the study, all participants were informed of its purpose, and their consent was acquired.

### Statistical Analysis

Using Microsoft Excel (Microsoft Corp) and Statistical Package for Social Sciences software (version 26; IBM Corp), the gathered data were analyzed. Factors related to the administration technique were evaluated using multivariate binary logistic regression to understand their independent impact. The decision tree analysis was done using Chi-square automatic interaction detector to explore hierarchical relationships and interactions among predictors of INCS counseling proficiency and to complement the findings of binary logistic regression. When *P*<.05 and the confidence level was 95%, it was deemed statistically significant.

### Ethical Considerations

Ethical approval reference number 210 (6-11) E2, 080/081, was provided by the institutional review committee of the Institute of Medicine, Tribhuvan University, before the commencement of the study. Written informed consent was provided by participants before any data were collected from the study site (Multimedia Appendix 2). The identity of participants will not be revealed in any information that will be published or released to third parties. The participants were not compensated for this study.

## Results

### Participant Characteristics

Pharmacists’ professional and demographic traits are listed in Table 1. The study involved 365 registered pharmacists as participants. Of the 365 pharmacists, 216 (59.2%) were ≤26 years old, and 239 were men (65.5%). In addition, 244 (66.8%) were single. Only 110 participants (30.1%) had a BPharm degree or above, whereas 255 (70%) had a DPharm degree. Moreover, 267 participants (73.2%) were early career (1‐4 y), whereas 98 (26.8%) were mid-career or late career (5 y and above). In all, 194 participants (53.2%) reported counseling patients on intranasal corticosteroids 1 to 4 times per week, but only 30 participants (8.2%) acknowledged any formal training in INCS administration. Additionally, only 75 participants (20.5%) used leaflets to counsel the patients.

**Table 1.** Demographic and professional characteristics of pharmacists (N=365).

Variables	Frequency	Percentage
Sex		
Male	239	65.5
Female	126	34.5
Age		
≤26 years	216	59.2
>26 years	149	40.8
Marital status		
Unmarried	244	66.8
Married	121	33.2
Qualification		
DPharm	255	69.9
BPharm and above	110	30.1
Years of experience		
1‐4 years	267	73.2
5 years and above	98	26.8
Intranasal corticosteroid spray counseling (per week)		
Occasionally	119	32.6
1‐4 times	194	53.2
More than 4 times	52	14.2
Received training		
Yes	30	8.2
No	335	91.8
Use of information material		
Yes	75	20.5
No	290	79.5

### Administration Technique Adherence and Proficiency Level

Among 365 participating pharmacists, adherence to INCS administration steps varied widely, as shown in Table 2. High adherence (>80%) was observed in 4 basic steps: removing the dust cap, replacing the cap, shaking the spray, and holding the bottle upright. In addition, moderate adherence (40%‐80%) was noted for 3 steps: inhaling while spraying, finger positioning, and nozzle insertion. However, low adherence (<40%) was observed for 5 steps, of which 4 were critical: blowing the nose, pointing the nozzle away from the septum, exhaling through the mouth, proper head positioning, and wiping the nozzle after use.

**Table 2.** Performance of each administration step by pharmacists (N=365).

Step	Steps for the administration of intranasal corticosteroid spray	Frequency	Percentage
1	Shake the spray in a vertical plane	309	84.7
2	Remove the dust cap	365	100
3	Blow the nose (critical)	39	10.7
4	Hold the spray bottle, pointing the tip of the nozzle up with the hand	293	80.3
5	Place the index and middle finger on the pusher and the thumb at the bottom of the spray bottle	220	60.3
6	Put the tip of the nozzle in one nostril and close the other side	146	40
7	Maintain a neutral head position or slightly tilt the head forward (critical)	122	33.4
8	Point the tip slightly outward, away from the septum (critical)	36	9.9
9	Squirt the spray into the nose while breathing in (critical)	287	78.6
10	Breathe out through the mouth (critical)	43	11.8
11	Wipe the nozzle with a tissue or handkerchief	123	33.7
12	Replace the cap	359	98.4

The participants’ median score across all 12 steps was 6. However, the 5 crucial steps only had a mean score of 1.9 (SD 1.09). Twelve points were awarded for completing all INCS counseling steps, of which 5 points were awarded for the 5 critical steps. Just 22 participants (6%) were able to accurately complete all 5 critical steps. We found that 193 (52.9%) of the registered pharmacists were inadequately knowledgeable on INCS patient counseling. Only 172 participants (47.1%) had adequate knowledge of INCS counseling.

### Factors Associated With Proper Administration Technique

Several professional and sociodemographic factors were shown to be substantially correlated with the degree of administration technique proficiency by the multivariate binary logistic regression analysis (Table 3). Years of experience, training, and marital status did not show statistically significant relationships, while sex, age, qualification, frequency of patient counseling weekly, and the utilization of information material were found to be significant predictors.

The likelihood of male pharmacists exhibiting proper technique was about 2 times higher than that of female pharmacists (*P*=.02). The probability of using an inappropriate INCS counseling technique was 89% lower for individuals who were older than 26 years (*P*=.001). Proficiency was substantially predicted by having used educational materials. Pharmacists who used educational materials were 96% less likely to perform inadequately (*P*=.005). Pharmacists with a BPharm degree or higher were also around 97% less likely to counsel inappropriately than those with a DPharm (*P*<.001). According to this study, individuals who advise patients on INCS 1‐4 times per week were 11 times more likely to demonstrate proficiency as opposed to those who counsel occasionally (*P*=.002).

**Figure 1.**
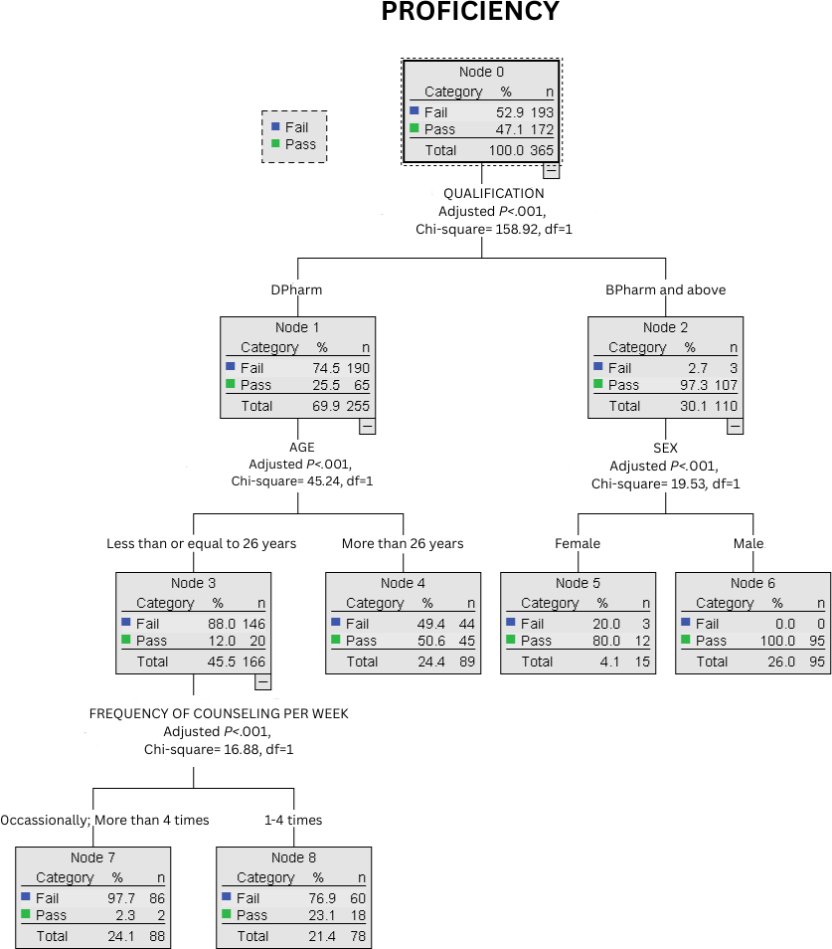
Classification tree model of predictors for proficient intranasal corticosteroid counseling among pharmacists.

The classification tree (Chi-square automatic interaction detector method), as shown in Figure 1, was developed to identify key predictors of pharmacist proficiency in INCS counseling. The final pruned classification included 5 levels with 9 terminal nodes, achieving an overall classification accuracy of 81.6%. The root node shows the entire study population, and subsequent splits identify variables that best differentiate proficiency levels. Terminal nodes represent final subgroups, displaying the proportion of pharmacists classified as proficient or nonproficient within each subgroup.

**Table 3.** Binary logistic regression analysis of proficiency level of administration technique and different sociodemographic and professional details variables.

Variable	Adjusted odds ratio	95% CI	*P*
Sex			
Male	2.30	1.11‐4.75	.02
Female	Reference		
Age			
≤26 years	0.11	0.03‐0.41	.001
>26 years	Reference		
Marital status			
Unmarried	2.39	0.71‐8.06	.16
Married	Reference		
Training			
No	—^a^	—	>.99
Yes	Reference		
Use of educational leaflet			
No	0.04	0.004‐0.38	.005
Yes	Reference		
Qualification			
DPharm	0.03	0.007‐0.14	<.001
BPharm and above	Reference		
Years of experience			
1‐4 years	0.80	0.33‐1.94	.62
5 years and above	Reference		
Intranasal corticosteroid spray counseling (weekly)			
Occasionally	4.80	0.91‐25.30	.06
1-4 times	11.21	2.35‐53.53	.002
>4 times	Reference		

a Not applicable.

The first and the most significant split was based on the participants’ educational qualifications. Only 65 of 255 (pass rate: 25.5%) pharmacists with a DPharm degree had adequate proficiency. However, of 110 pharmacists with a BPharm degree or higher, 107 had adequate proficiency (pass rate: 97.3%). Among the DPharm group, age was another significant predictor. Among those aged less than 26 years, only 20 of 166 participants (12%) had adequate proficiency, whereas among the older peers, 45 of 89 had adequate proficiency. Similarly, among the BPharm group, another major factor was gender. Male pharmacists were found to be 100% proficient in INCS counseling, with all 95 participants demonstrating adequate proficiency, whereas only 12 of 15 female participants had adequate proficiency.

Finally, for younger DPharm degree holders (≤26 y old), the frequency of INCS counseling was another predictor. Those younger participants who counseled occasionally or more than 4 times per week had significantly lower proficiency (2/88 had adequate proficiency) compared to those who counseled 1-4 times per week (18/78 had adequate proficiency).

## Discussion

### Principal Findings

This study addresses a critical gap in pharmacist competency regarding INCS within resource-constrained health systems, where pharmacists are front-line care providers. This study is among the first in Nepal to assess pharmacists’ proficiency with INCS counseling. The survey revealed a significant gap in the participants’ understanding of INCS counseling, which helps in understanding its impact on the health outcomes of patients. Approximately 50% of the pharmacists lacked adequate INCS counseling abilities. According to this study, only 6% of pharmacists were able to complete all the essential patient counseling steps that are crucial for appropriate drug administration and to minimize the risk of adverse drug reactions. Classification tree analysis showed that educational degree was the primary predictor of INCS counseling proficiency. Those with BPharm degrees or higher were far more proficient than DPharm degree holders.

The survey’s conclusions about the inadequate INCS administration abilities of Nepali registered pharmacists are in line with the findings of patients and medical professionals worldwide [2,14,26]. Only 22 of 365 of pharmacists (6%) performed all recommended steps correctly, which was similar to a study of health care workers in Thailand [14]. However, even in a developed country like the Netherlands, it was found that only about 36% of health care workers were able to complete all the critical steps [26]. These observations suggest that there is a major gap in skill related to INCS counseling across nations, rather than it being a local issue. Due to this inadequate proficiency among pharmacists, there is a high risk of an increase in adverse drug reactions in patients. Therefore, the educational system must be improved to include simulation-based training and mandatory hands-on workshops that allow students and professionals to practice essential steps repeatedly and understand their rationale.

The high proportion of pharmacists demonstrating steps 1, 2, 4, and 12 correctly (>80%) likely reflects common-sense knowledge (shake, remove dust cap, hold the bottle, replace the cap) that is often taught in basic therapy discussions. However, steps like bending the head forward or cleaning the nozzle were rarely done correctly (<40%). This may cause improper drug disposition, irritation in the throat, and increased risk of contamination [27]. Similarly, only about 10% of participants were counseled about pointing the nozzle away from the nasal septum, which reduces the risk of nasal irritation, dryness, and epistaxis, and improves drug absorption from the lateral nasal wall [12,27]. In addition, the steps necessary to remove mucus or debris or obstruction from the nose and reduce throat irritation (ie, blowing the nose before use and exhaling through the mouth) were only performed by about 10% of participants [12]. Patients who are not taught to clean the spray tip may experience clogging or contamination.

These differences align with prior studies indicating that procedural complexity and a lack of continuing pharmacy education (CPE) or training contribute to inconsistent adherence to medical device protocols [28]. This study highlights that even pharmacists, who are trained professionals, often lack full mastery of device use and suggests there is a need to improve the pharmacy curriculum and landscape of CPE in Nepal.

One of the important differences was the pharmacist’s qualification. BPharm graduates were about 97% less likely to demonstrate incorrect technique than DPharm graduates. The latter finding reflects the differences in Nepal’s educational system. The 3-year DPharm program in Nepal has traditionally emphasized dispensing skills, whereas the BPharm and PharmD curricula include more clinical training.

Shrestha et al [29] found that Nepal’s conventional pharmacy education is mostly lecture-based and industry-oriented, with limited practical training in hospitals. Bhuvan et al [30] also documented the challenges in transitioning to PharmD in Nepal, with a focus on patient care and pharmaceutical care. This highlights a need for a gradual change in current policy. Medical devices training should be included in the DPharm degree, and seminars and workshops should involve DPharm students and graduates. Pharmacy regulators in Nepal, such as the Nepal Pharmacy Council or the DDA, may consider upgrading community pharmacists’ credentials or introducing minimum competency assessments for patient counseling.

In this study, it was found that pharmacists who used educational leaflets were much more proficient. This is similar to findings of other studies where pharmacist-led interventions with practical demonstrations and the use of leaflets dramatically improved patient technique [25,31]. These educational leaflets significantly reduce the cognitive load of pharmacists and ensure the completeness of all steps. These aids also engage patients through teach-back, reinforce learning beyond completeness, and boost the pharmacist’s confidence and professionalism. Therefore, pharmacists should be encouraged to use educational leaflets during counseling sessions on INCS use.

In our study, increasing age (>26 y) was significantly associated with improved INCS counseling proficiency. A study conducted in Korea also found that proficiency in patient counseling regarding topical corticosteroids significantly improved with increasing age [32]. Thus, suggesting increased clinical exposure, more trainings, mature communication skills, and more frequent patient interaction may contribute to better proficiency. In order to succeed in INCS counseling, younger pharmacists must receive sufficient training throughout their time in pharmacy school. They should also attend workshops on medical devices, communication techniques, and patient counseling.

Interestingly, participants counseling on INCS use 1‐4 times per week have a much higher proficiency (almost 11 times higher) compared with that of participants counseling only occasionally. This relationship likely reflects that a moderate counseling volume provides sufficient repetition to hone skills and confidence, while excessive patient load and task interruptions may reduce time for careful demonstration and feedback [33,34]. Simulation training could help low-counseling pharmacists achieve similar proficiency without relying on clinical exposure.

The analysis of this survey revealed that, among BPharm graduates, males have about 2 times higher odds of proficiency than females regarding INCS counseling. However, the existing literature does not present any conclusive or consistent evidence of sex-based differences in nasal spray or inhalation administration technique among pharmacists. Therefore, the observed difference may reflect contextual, educational, or practice-related factors rather than true gender-based differences.

In our study, 335 of 365 pharmacists (91.8%) lacked specific training. This suggests that continuing professional development for pharmacists in Nepal is sorely needed. According to a recent analysis of continuing professional development in Nepal, CPE is still in its infancy; therefore, working pharmacists are not informed of the latest treatments or best practices [35]. Establishing regular INCS technique workshops or integrating device training into the curriculum could narrow the gap. Given pharmacists’ accessibility in rural and urban Nepal [36,37], such measures could rapidly propagate correct practice.

Pharmacists’ poor INCS technique skills are concerning but can be resolved. Targeted training in Nepal could help pharmacists improve their skills quickly. Emphasizing AR and device technique in undergraduate pharmacy programs and requiring competency demonstrations during examinations could have a lasting impact. In addition, public health campaigns might encourage patients to ask pharmacists for a demonstration of INCS technique. In the long term, strengthening pharmacy education and integrating pharmacists into asthma/allergy care pathways will benefit Nepal’s health care system by improving primary-level management of chronic respiratory diseases.

### Limitations

This study was conducted in urban Kathmandu, so findings may not generalize to rural areas, where pharmacies are fewer and mainly operated by trained dispensers. This study has a cross-sectional design, so it cannot prove causality. Potential confounders like workload details were not measured, which may have partly contributed to the large adjusted odds ratio of some predictors. Some of the findings may be extreme due to small subgroups such as pharmacists who had received formal training or those providing frequent INCS counseling. A small sample count can result in unstable estimates and inflate the results. In addition, model overfitting can occur due to the inclusion of multiple interrelated predictors during logistic regression. This study used a small sample size for the reliability test and only used an expert-based face validity test, which may limit the robustness and generalizability of the study. Even with anonymized, behavior-focused questions, self-reported variables like the frequency of counseling and the usage of educational leaflets may be overestimated due to recall and social desirability bias, especially in in-person interviews. This shortcoming is highlighted and the necessity of objective assessment is supported by the observed difference between overall self-reported sufficiency and inadequate performance on critical steps. We recommend a weighted or competency-based scoring model in future studies. Finally, the presence of an interviewer might have influenced the participants’ performance (ie, the Hawthorne effect), possibly inflating technique scores. However, due to the low proficiency observed among the participants, any such effect was limited.

### Conclusion

This study highlights that more than half of the participants did not have adequate skills to demonstrate proper INCS usage technique. This can lessen its effectiveness in treating AR and increase the likelihood of adverse drug reactions in patients, such as dyspnea, anosmia, ageusia/dysgeusia, epistaxis, and headache. The lack of knowledge is mainly due to poor exposure to this topic in pharmacy school. In addition, training and seminars are limited both during school and after registration as a pharmacist. Resolving this problem should be one of the most important tasks for the Nepal Pharmacy Council and the Health Ministry as AR is very common in Nepal. Upgrading pharmacy curricula, mandating continuing education, and providing standardized counseling materials may empower pharmacists to counsel patients on the correct technique.

## Supplementary material

10.2196/83042 Multimedia Appendix 1 Sensitivity analysis.

10.2196/83042 Multimedia Appendix 2 Informed consent form.

## Abbreviations

AR: allergic rhinitis
BPharm: Bachelor of Pharmacy
CPD: continuing professional development
CPE: continuing pharmacy education
DDA: Department of Drug Administration
DPharm: Diploma in Pharmacy
INCS: intranasal corticosteroid spray
PharmD: Doctor of Pharmacy
STROBE: Strengthening the Reporting of Observational Studies in Epidemiology

## References

[1] García-Almaraz R, Reyes-Noriega N, Del-Río-Navarro BE (2021). Prevalence and risk factors associated with allergic rhinitis in Mexican school children: Global Asthma Network Phase I. World Allergy Organ J.

[2] Akhouri S, House SA, Doerr C Allergic Rhinitis (Nursing).

[3] Nepali R, Sigdel B, Dubey T, Kc N, Gurung B, Baniya P (2022). Common allergens in patients of allergic rhinitis in Gandaki province of Nepal. JGMC Nepal.

[4] Moitra S, Mahesh PA, Moitra S (2023). Allergic rhinitis in India. Clin Experimental Allergy.

[5] Sinha B, Singla R, Chowdhury R, Vibha (2015). Allergic rhinitis: a neglected disease - a community based assessment among adults in Delhi. J Postgrad Med.

[6] Bauchau V, Durham SR (2004). Prevalence and rate of diagnosis of allergic rhinitis in Europe. Eur Respir J.

[7] Allergic rhinitis: practice essentials, background, pathophysiology. Medscape.

[8] Klimek L, Bachert C, Pfaar O (2019). ARIA guideline 2019: treatment of allergic rhinitis in the German health system. Allergo J Int.

[9] Neffen H, Wingertzahn MA (2010). Ciclesonide, a hypotonic intranasal corticosteroid. Allergy Asthma Proc.

[10] Ahsanuddin S, Povolotskiy R, Tayyab R (2021). Adverse events associated with intranasal sprays: an analysis of the food and drug administration database and literature review. Ann Otol Rhinol Laryngol.

[11] Ganesh V, Banigo A, McMurran AEL, Shakeel M, Ram B (2017). Does intranasal steroid spray technique affect side effects and compliance? Results of a patient survey. J Laryngol Otol.

[12] Rattanawong S, Wongwattana P, Kantukiti S (2022). Evaluation of the techniques and steps of intranasal corticosteroid sprays administration. Asia Pac Allergy.

[13] Benninger MS, Hadley JA, Osguthorpe JD (2004). Techniques of intranasal steroid use. Otolaryngol Head Neck Surg.

[14] Rollema C, van Roon EN, de Vries TW (2019). Inadequate quality of administration of intranasal corticosteroid sprays. J Asthma Allergy.

[15] Gurung U, Khadgi S (2024). Intranasal corticosteroid spray usage in patients with allergic rhinitis: correctness in technique and compliance. J Inst Med Nepal.

[16] Al-Taie A (2025). A systematic review for improper application of nasal spray in allergic rhinitis: a proposed role of community pharmacist for patient education and counseling in practical setting. Asia Pac Allergy.

[17] STROBE - Strengthening the reporting of observational studies in epidemiology.

[18] Elm E von, Altman DG, Egger M, Pocock SJ, Gøtzsche PC, Vandenbroucke JP (2007). Strengthening the reporting of observational studies in epidemiology (STROBE) statement: guidelines for reporting observational studies. BMJ.

[19] Search pharmacy. Department of Drug Administration, Ministry of Health and Population, Government of Nepal.

[20] Sample size calculator. Raosoft Inc.

[21] Nepal Pharmacy Council.

[22] Rollema C, van Roon EN, van Boven JFM (2022). Pharmacology, particle deposition and drug administration techniques of intranasal corticosteroids for treating allergic rhinitis. Clin Experimental Allergy.

[23] How and when to use mometasone nasal spray. NHS.

[24] Rollema C, van Roon EM, Schilder AG, de Vries TW (2019). Evaluation of instructions in patient information leaflets for the use of intranasal corticosteroid sprays: an observational study. BMJ Open.

[25] Kc B, Khan GM, Shrestha N (2020). Nasal spray use technique among patients attending the out-patient department of a tertiary care hospital, Gandaki Province, Nepal. Integr Pharm Res Pract.

[26] de Boer M, Rollema C, van Roon E, Vries T de (2020). Observational study of administering intranasal steroid sprays by healthcare workers. BMJ Open.

[27] Intranasal spray technique. National Asthma Council Australia.

[28] Bosnic-Anticevich SZ, Sinha H, So S, Reddel HK (2010). Metered-dose inhaler technique: the effect of two educational interventions delivered in community pharmacy over time. J Asthma.

[29] Shrestha S, Shakya D, Palaian S (2020). Clinical pharmacy education and practice in Nepal: a glimpse into present challenges and potential solutions. Adv Med Educ Pract.

[30] Bhuvan KC, Subish P, Mohamed Izham MI (2011). PharmD education in Nepal: the challenges ahead. Am J Pharm Educ.

[31] Chew CC, Lim XJ, Letchumanan P, George D, Rajan P, Chong CP (2024). The effectiveness of pharmacist-led educational model in adult patients with allergic rhinitis: a single-center randomized control trial protocol (AR-PRISE RCT). Trials.

[32] Kang MJ, Park JH, Park S (2020). Community pharmacists’ knowledge, perceptions, and practices about topical corticosteroid counseling: a real-world cross-sectional survey and focus group discussions in Korea. PLoS ONE.

[33] Shao SC, Chan YY, Lin SJ (2020). Workload of pharmacists and the performance of pharmacy services. PLoS One.

[34] Lea VM, Corlett SA, Rodgers RM (2012). Workload and its impact on community pharmacists’ job satisfaction and stress: a review of the literature. Int J Pharm Pract.

[35] Khatiwada AP, Shrestha S, Sapkota B (2022). Continuing pharmacy education: exploring the status and future prospects in Nepal. Adv Med Educ Pract.

[36] Lourenço O, Bosnic-Anticevich S, Costa E (2020). Managing allergic rhinitis in the pharmacy: an ARIA guide for implementation in practice. Pharmacy (Basel).

[37] Ikhile I, Anderson C, McGrath S, Bridges S (2018). Is the global pharmacy workforce issue all about numbers?. Am J Pharm Educ.

[38] Study dataset. Zenodo.

