# Supplementary material for: Administration Technique of Intranasal Corticosteroid Sprays Among Nepali Pharmacists: Cross-Sectional Study
Source: JMIRx Med. 2026 Jan 29;7:e83042. doi: 10.2196/83042 (PMC12855720; doi:10.2196/83042)
Supplement: Multimedia Appendix 1 [file xmed-v7-e83042-s001.docx]

**Sensitivity Analysis carried out for different cut off using binary Logistic Regression**

| **Variable** | **Cutoff >6** | | **Cutoff >5** | | **Cutoff >7** | |
| --- | --- | --- | --- | --- | --- | --- |
|  | **aOR (CI)** | **P-value** | **aOR (CI)** | **P-value** | **aOR (CI)** | **P-value** |
| **Sex** | | | | | | |
| Male | 2.30 (1.11 – 4.75) | **0.025** | 2.590  (1.385 - 4.844) | **0.003** | 5.657  (1.374 -23.298) | **.016** |
| Female | **Ref** | | |  |  |  |
| **Age** | | | | | | |
| less than or equal to 26 years | 0.11 (0.03 – 0.41) | **0.001** | 0.136 (0.040- .467) | **0.002** | 1.266  (0.171 -9.378**)** | .818 |
| More than 26 years | **Ref** | | |  |  |  |
| **Marital Status** | | | | | | |
| Unmarried | 2.39 (0.71 – 8.06) | 0.160 | 3.123 (0.99 -9.849) | .052 | 1.210  (0.265 -5.524) | .806 |
| Married | **Ref** | | |  |  |  |
| **Training** | | | | | | |
| No | - | 0.998 | - | .996 | - | .995 |
| Yes | **Ref** | | |  |  |  |
| **Use of Information Material** | | | | | | |
| No | 0.04 (0.004 – 0.38) | **0.005** | **-** | .996 | 0.792 (0.027 - 23.435) | .893 |
| Yes | **Ref** | | |  |  |  |
| **Qualification** | | | | | | |
| Dpharm | 0.03 (0.007 – 0.14) | **0.000** | .148  (0.041 -0.541) | **.004** | **-** | .993 |
| BPharm and above | **Ref** | | |  |  |  |
| **years of experience** | | | | | | |
| 1-4 years | 0.80 (0.33 – 1.94) | 0.625 | .944  (0.415 -2.149) | .891 | **-** | .995 |
| 5 years and above | **Ref** | | |  |  |  |
| **INCS counseling (weekly)** | | | | | | |
| Occasionally | 4.80 (0.91 – 25.3) | 0.064 | .512  (0.166 -1.582) | .245 | 0.776 (0.113 -5.320) | .796 |
| 1-4 times | 11.21 (2.35 – 53.53) | **0.002** | 0.978  (0.341 -2.810) | .967 | 1.183 (0.241 - 5.802) | .836 |
| Above 4 times | **Ref** | | |  |  |  |
