# Supplementary material for: Administration Technique of Intranasal Corticosteroid Sprays Among Nepali Pharmacists: Cross-Sectional Study
Source: JMIRx Med. 2026 Jan 29;7:e83042. doi: 10.2196/83042 (PMC12855720; doi:10.2196/83042)
Supplement: Multimedia Appendix 2 [file xmed-v7-e83042-s002.pdf]

INFORMED CONSENT FORM

Administration Technique of Intranasal Corticosteroid Sprays among Nepali Pharmacists: A Cross-Sectional Study

Department of Pharmacy, Institute of Medicine, Tribhuvan University, Kathmandu, Nepal

I, …………………………………………, male/female of ……….. years age, hereby confirm that I have read and understood the information sheet and consent form for this research being conducted by …………………………………………, and have had the opportunity to ask questions about it. I hereby declare that,

1. I understand that my participation in the study is voluntary and that I am free to withdraw at any time, without giving any reason, without my medical care or legal rights being affected.

2. I understand that the researchers, the IRC and other regulatory authorities will not need my permission to look at my health records both in respect of the current study and any further research that may be conducted in relation to it, even if I withdraw from the trial. I agree to this access. However, I understand that my identity will not be revealed in any information that will be published or released to the third parties.

3. I agree not to restrict the use of any data or results that arise from this study provided that such use is only for scientific purpose(s).

4. I agree to take part in this study.

Signature (or Thumb impression) of the research participant/Legal Guardian

Signature: ……………………………

Name: …………………………………………

Date: ……………………………

Investigator's Signature: ……………………………

Name: ……………………………………….. ………………………… Date: …………………………….

Signature (or Thumb impression) of Witness Signature: ……………………………

Name: …………………………………………

Date: ……………………………
